# Clinical false positives resulting from recent intravenous immunoglobulin therapy: case report

**DOI:** 10.1186/s12879-021-05986-z

**Published:** 2021-03-21

**Authors:** Janarthanee Suresh, Barry D. Kyle

**Affiliations:** Department of Pathology and Laboratory Medicine, College of Medicine, University of Saskatchewan, Saskatoon, S7N 0W8 Canada

**Keywords:** IVIg therapy, Serology, Hepatitis, Cytomegalovirus, Thrombocytopenia

## Abstract

**Background:**

Many clinicians are aware that certain therapies administered to their patients can have downstream consequences in the form of clinical laboratory test interferences. This is particularly true of laboratory tests that depend on, or directly involve the use of, antibody-based methodology. Intravenously-administered immunoglobulin therapy is one such treatment that can in theory directly impact the results of particular tests in the area of viral serology. This study can help serve as a reference for clinicians researching the impact of intravenously-administered immunoglobulin therapy in the context of positive results that do not reflect the clinical background of the patient.

**Case presentation:**

We describe a case whereby an intravenously-administered immunoglobulin therapy led to a series of clinical false positives in viral serology, inconsistent with the known patient history as well as recent laboratory results. The patient presented to hospital with petechiae-type bleeding rashes and was investigated for thrombocytopenia after initial blood investigations indicated very low platelets.

Subsequent testing of the potential causes for low-platelet involved several viral serology investigations, including hepatitis, cytomegalovirus and human immunodeficiency virus. Initial testing indicated patient exhibited negative status for all viral antibodies and antigens (except immunity for hepatitis B surface antigen antibody).

As part of the thrombocytopenia treatment, intravenously-administered immunoglobulin therapy was administered, and subsequent viral serology was ordered. These investigations indicated a positive status for several hepatitis antibodies as well as cytomegalovirus.

**Conclusions:**

This case study illustrates the potential for improper diagnosis of previous or ongoing infection status in patients administered IVIg therapy. Caution should be exercised particularly when interpreting results involving cytomegalovirus and hepatitis.

## Background

For the past two to three decades, intravenous immunoglobulin (IVIg) therapy has been a relatively common treatment for autoimmune diseases. IVIg therapy is often indicated for either primary or secondary immunodeficiency states [1]. The manufacturing of IVIg preparations for human use generally involves the pooling of several thousand plasma donors, with the subsequent removal of the majority of IgM and IgA fractions, with the bulk of the remaining fraction consisting mainly of IgG immunoglobulins [2, 3].

Health Canada has approved the use of IVIg therapy for primary immune deficiencies and idiopathic thrombocytopenic purpura (ITP). IVIg therapy is also indicated for secondary immune deficiencies such as allogenic bone transplantation, B-cell chronic lymphocytic leukemia and pediatric human immunodeficiency (HIV) infection [4]. Additionally, off-label use of IVIg therapy is encountered for other pathologies, such as multiple sclerosis (MS). Documented adverse events following IVIg therapy include but are not limited to pyrexia, acute renal failure, headache, nausea and hepatitis C [5]. However the adverse reactions are typically mild [1, 6].

IVIg therapy preparations contain pools of IgG proteins from wide swathes of the population. Many clinical laboratory tests are designed to detect pathogen-specific types of IgG immunoglobulins (e.g. hepatitis A antibody test) in patient’s blood samples. With this in mind, there is a potential for the laboratory test to detect and report the presence of IgG proteins that originate from the IVIg preparation, and not necessarily IVIg generated by the patient’s own immune system production. Indeed, IVIg preparation manufacturers (e.g. Gammagard) specifically mention the potential for direct and indirect false positive serologic test results following therapy in their prescribing information documents. Here, we present a case whereby a patient presented to hospital with thrombocytopenia, was checked for viral infection markers and found to be negative, and was then administered IVIg therapy. Additional viral serology testing, ordered in error later that day, indicated a change in viral immunity status and prompted an investigation. Patient consent was obtained in accordance with the guidelines of the Saskatchewan Health Authority.

## Case presentation

A 34 year old female was admitted to the Royal University Hospital, Saskatchewan, after noticing petechiae on her legs and inside of her mouth. The complete blood count (CBC) was remarkable for demonstrating particularly thrombocytopenia (4x10e9/L, RI 150–400 x10e9/L) and lymphopenia (0.88x10e9/L, RI 1.5–4.0x10e9/L). Hemoglobin concentration (141 g/L, RI 110–160 g/L) was normal. The patient had a history of multiple sclerosis (MS), and management with Alemtuzumab™ monoclonal antibody therapy, most recently administered (36 mg over three days) approximately two months prior to patient admission. She was investigated and treated for thrombocytopenia.

Blood was drawn for viral serology testing that included cytomegalovirus (CMV) IgM and IgG antibodies, hepatitis virus antibodies (hepatitis A total, hepatitis A IgM, hepatitis B surface IgG, hepatitis B core IgM, hepatitis B total, hepatitis C), hepatitis B surface antigen, and HIV investigations. All serologies were performed on an automated Roche Diagnostics e601 platform analyzer according to manufacturer’s instructions. Serology test results indicated that the patient had immunity status for hepatitis B surface IgG antibody (i.e. > 1000 IU/L). However, the patient did not exhibit immunity or presence of the other hepatitis markers tested (Table 1, Pre-IVIg). These results were consistent with the patient history over the course of several years. The patient had no prior evidence of blood borne disease, having been reported negative for CMV, HIV and hepatitis panel testing, although the patient did have positive hepatitis B surface antibody test results, consistent with the prior immunization for hepatitis B in 2018.

**Table 1:** Results of patient testing before and after IVIg administration, discrepant results noted in bold

Investigation	Reference intervals	Pre-IVIg		Post IVIg	
	(units)	Interpretation	Value	Interpretation	Value
Total IgA	0.4–3.5 (g/L)	Normal	1.16	Normal	1.1
Total IgG	6.5–16.0 (g/L)	Normal	10.01	**High**	**36.93**
Total IgM	0.5–3.0 (g/L)	Normal	0.63	Normal	0.59
CMV IgG	< 0.5 (U/mL)	Negative	< 0.3	**Positive**	**467**
Hepatitis A total antibody	< 20.0 (IU/L)	Negative	8.0	**Positive**	**> 60**
Hepatitis A IgM antibody	< 1.0 (COI)	Negative	< 0.3	Negative	< 0.3
Hepatitis B surface IgG antibody	< 10.0 (IU/L) ^a^	Positive	> 1000	Positive	> 1000
Hepatitis B core IgM antibody	< 1.0 (COI)	Negative	0.1	Negative	0.1
Hepatitis B core total antibodies ^b^	> 1.0 (COI)	Negative	2.3	**Positive**	**< 0.8**
Hepatitis B surface antigen	< 0.9 (COI)	Negative	0.3	Negative	0.3
Hepatitis C antibody	< 0.9 (COI)	Negative	0	Negative	0.1

^a^ = non-immune ^b^ = competitive immunoassay; results > 1.0 are negative

The patient was administered 20 g of IVIg (Gammagard™ 20 g) as part of the treatment plan for thrombocytopenia.

Additional serology was ordered in error after the IVIg administration and generated discrepant results (Table 1, Post IVIg, bold), prompting an investigation by laboratory medical staff. Repeat testing on the initial specimen demonstrated the same result initially reported. Similarly, the post-IVIg specimen was retested, and the result was consistent. Additional blood draws consistently demonstrated the apparent newly-positive results for viral serology. Plasma samples drawn prior to and following the IVIg therapy were also investigated. These specimens were measured for total IgA, IgG and IgM immunoglobulins, and demonstrated a substantial increase in the IgG component (Table 1), consistent with expected results following treatment with 20 g of IVIg. Neither HIV testing, nor hepatitis C testing interpretation were changed by IVIg administration (data not shown), which is consistent with donor screening-out criteria by the manufacturer. Similarly, CMV IgM status was negative pre- and post-IVIg administration (data not shown).

## Discussion and conclusions

This case highlights the potential for false positive serological test results after IVIg administration. Several other studies have noted changes in apparent immune status following transfusion products, which typically decrease as a function of immunoglobulin half-life. There is a distinct risk of inappropriate follow-up testing and investigations arising out of apparent positive serology results (e.g. CMV) [3, 7, 8].

The passive transfer of anti-hepatitis B antibodies from IVIg products and its consequences on clinical trials and treatments has been documented [3]. Although this case study was restricted to only hepatitis and CMV serology testing discrepancies, it is possible that other markers of past infection could have been similarly affected. The passive transmission of anti-troponemal antibody and anti-*Toxoplasma gondii* antibodies have also been reported and studied [7, 8].

Furthermore relapsing-remitting MS patients are often treated with Alemtuzumab. This humanized monoclonal antibody targets CD52 proteins, which are expressed in all leukocytes. The observed therapeutic effects lasts long after treatment discontinuation [9, 10]. Aguirre, et al. has reported on cases of CMV reactivation after Alemtuzumab treatment, with recommendation of constant vigilance beyond first therapy [10]. CMV result interpretation can also be further complicated by additional clinical factors (e.g. subclinical hemolysis with IVIg therapy.) [11]

Immune globulin preparations collectively represent thousands of plasma donor’s immune status [12], with significant lot-to-lot variability. Additionally, proprietary differences occur in manufacturing methods and the unique tolerability and safety profile of each IVIg product may vary [5]. Serology testing for various infectious diseases are frequently requested without the knowledge of previous IVIg exposure [13]. Hemolysis and false positive serologic test results are sometimes unexpected consequences of IVIg therapy [14, 15]. False positives may occur as a result of reactivity of the plasma donor IgG proteins to the test reagents [16]. Interpretation of such results should be done with caution [13]. It is recommended to avoid serological testing in patients undergoing IVIg therapy. Following the therapy, it is possible, if not likely, that continued false-positive results will occur for several days and perhaps weeks, and will vary as a function of the status of IgG half-life. This will be influenced by several factors including both the IgG donor and recipient patient profiles, as well as issues pertaining to storage/stability, and manufacturer preparation constitution (e.g. proprietary additives). Clinicians should carefully consult the prescribing information on IVIg therapy documentation, which clearly warns about the potential for misinterpretation for serological testing as well as other interferences (e.g. direct or indirect antiglobulin tests), which is regular clinical practice [17].

Finally, although not directly observed in this case, clinicians may misinterpret hepatitis surface antibody positivity as legitimate immunity in patients undergoing IVIg therapy. Such testing is a requirement for various professions, particularly in health care (e.g. nurse), that mandate that immune status is established.

## Data Availability

Not applicable.

## Abbreviations

IVIg: Intravenous immunoglobulin
CMV: Cytomegalovirus
HIV: Human immunodeficiency virus
IgA: Immunoglobulin A
IgG: Immunoglobulin G
IgM: Immunoglobulin M
CBC: Complete blood count
IU/L: International units per litre
U/mL: Units per millilitre
COI: Cut-off index
RI: Reference interval
MS: Multiple sclerosis
